# Construction and application of the genome-scale metabolic model of *Streptomyces radiopugnans*

**DOI:** 10.3389/fbioe.2023.1108412

**Published:** 2023-02-17

**Authors:** Zhidong Zhang, Qi Guo, Jinyi Qian, Chao Ye, He Huang

**Affiliations:** ^1^ College of Biotechnology and Pharmaceutical Engineering, Nanjing Technology University, Nanjing, China; ^2^ Institute of Microbiology, Xinjiang Academy of Agricultural Sciences, Urumqi, China; ^3^ School of Food Science and Pharmaceutical Engineering, Nanjing Normal University, Nanjing, China

**Keywords:** geosmin, *Streptomyces radiopugnans*, genome-scale metabolic model, culture condition optimization, metabolic engineering

## Abstract

Geosmin is one of the most common earthy-musty odor compounds, which is mainly produced by *Streptomyces*. *Streptomyces radiopugnans* was screened in radiation-polluted soil, which has the potential to overproduce geosmin. However, due to the complex cellular metabolism and regulation mechanism, the phenotypes of *S. radiopugnans* were hard to investigate. A genome-scale metabolic model of *S. radiopugnans* named *i*ZDZ767 was constructed. Model *i*ZDZ767 involved 1,411 reactions, 1,399 metabolites, and 767 genes; its gene coverage was 14.1%. Model *i*ZDZ767 could grow on 23 carbon sources and five nitrogen sources, which achieved 82.1% and 83.3% prediction accuracy, respectively. For the essential gene prediction, the accuracy was 97.6%. According to the simulation of model *i*ZDZ767, D-glucose and urea were the best for geosmin fermentation. The culture condition optimization experiments proved that with D-glucose as the carbon source and urea as the nitrogen source (4 g/L), geosmin production could reach 581.6 ng/L. Using the OptForce algorithm, 29 genes were identified as the targets of metabolic engineering modification. With the help of model *i*ZDZ767, the phenotypes of *S. radiopugnans* could be well resolved. The key targets for geosmin overproduction could also be identified efficiently.

## Introduction

Geosmin (trans-1,10-dimethyl-trans-9-decalol) is an irregular sesquiterpenoid of various actinomycetes and fungi, which has a distinct earthy or musty odor (Jiang et al., 2007). Geosmin is associated with the flavors in drinking water, wine, fish, and other foodstuffs. Several microorganisms, such as most *Streptomyces* (Jiang et al., 2006; Becher et al., 2020) and several species of cyanobacteria (Jiang et al., 2006; Giglio et al., 2008), myxobacteria (Dickschat et al., 2005), and fungi (Liato and Aider, 2017) can produce geosmin. *Streptomyces radiopugnans* belongs to the genus of *Streptomyces* and has been isolated from radiation-polluted soil from the Xinjiang Province in China (Mao et al., 2007). The genome of *S. radiopugnans* was sequenced in 2016 and can be accessed from the NCBI database. However, limited by the lack of experimental data, the regulation mechanism of geosmin biosynthesis was not clear in *S. radiopugnans*.

The genome-scale metabolic model (GSMM) is a mathematical model, which presents the gene-protein-reaction relationship. GSMM has been being developed for more than 20 years, since the first published GSMM of *Haemophilus influenzae* in 1999. Over 2,000 GSMMs have been published for over 1,000 organisms (Ye et al., 2022). With the increase of experimental data, some published GSMMs are continuously being improved. Some typical organisms, such as *Escherichia coli* K12 (6 GSMMs) (Ye et al., 2022), *Saccharomyces cerevisiae* S288c (12 GSMMs) (Ye et al., 2022), and *Yarrowia lipolytica* CLIB 122 (6 GSMMs) (Loira et al., 2012; Pan and Hua, 2012; Kavscek et al., 2015; Kerkhoven et al., 2016; Wei et al., 2017; Mishra et al., 2018) have more than one GSMM. Combined with different algorithms, GSMMs have been widely applied in network properties analysis, cellular phenotype prediction, metabolic engineering guidance, model-driven discovery, evolutionary process exploration, and interspecies interaction identification (Gu et al., 2019; Jansma and El Aidy, 2021; Patra et al., 2021).

In this study, based on the genome sequence of *S. radiopugnans*, a GSMM named *i*ZDZ767 was constructed. Model *i*ZDZ767 contained 1,411 reactions, 1,399 metabolites, and 767 genes. Compared with experimental data, *i*ZDZ767 could achieve 82.1% and 83.3% accuracy for the utilization of different carbon sources and nitrogen sources. In addition, the prediction accuracy of essential genes was 97.6%, compared with the DEG database (Luo et al., 2021). Then, based on *i*ZDZ767, D-glucose and urea were identified as the most suitable carbon source and nitrogen source, respectively. The experiments proved that when urea was used as nitrogen and controlled at 4 g/L, geosmin production could reach 581.6 ng/L. Finally, 29 genes (seven upregulation, six downregulation, and 16 knockout targets) were identified as potential targets, which could improve the geosmin synthesis rate using the OptForce algorithm (Ranganathan et al., 2010). This study provides new insights that could be used to investigate the phenotype of *S. radiopugnans* and identify the metabolic engineering targets for geosmin overproduction.

## Materials and methods

### Strain

The *S. radiopugnans* R97^T^ strain was screened from the contaminated radiation-contaminated area in Xinjiang, China (Mao et al., 2007).

### Genome sequence

The genome sequence of *S. radiopugnans* was downloaded from the NCBI database (https://www.ncbi.nlm.nih.gov/genome/49825?genome_assembly_id=1862392). The protein sequences of *S. radiopugnans* were downloaded from the UniProt database (https://www.uniprot.org/taxonomy/403935).

### Culture medium

The fermentation medium (1 L) contained 10 g/L glucose, 4 g/L yeast tract, 4 g/L K_2_HPO_4_, 4 g/L KH_2_PO_4_, and 0.5 g/L MgSO_4_. Before cultivation, the medium pH was adjusted to 7.2 using NH_4_OH (25%, v/v).

### Culture condition

For shake-flask cultivation, the *S*. *radiopugnans* strain was first cultured on an agar plate. Then, a single colony was selected to be cultured in 50 mL fresh medium until the OD_600_ reached a value of 0.8. Finally, the strain was transferred into a 500 mL shake-flask containing 50 mL fermentation medium and cultivated at 30°C for 240 h with shaking at 200 rpm.

### Determining the growth rate and glucose consumption rate

The optical density (OD) was first measured at 600 nm with a spectrophotometer. The cell dry weight was then calculated by multiplying OD_600_ by 0.36 g/L (Supplementary Figure S1) (Fischer and Sawers, 2013). The growth curve was fitted using the Logistic function of Origin software. Finally, the cell growth rate was calculated with differential values of cell dry weight (Supplementary Figure S2). Similarly, the glucose consumption rate was also calculated using Origin software, based on the experimental data (Supplementary Figure S3).

### Geosmin extraction and analysis

The extraction and analysis of geosmin were followed by (Shen et al., 2021).

### Prediction of optimized fermentation conditions

The robustness analysis [(controlFlux, objFlux) = robustnessAnalysis (model, controlRxn, nPoints, plotResFlag, objRxn, objType)] program was run in MATLAB to simulate the effect of the urea uptake rate on the synthesis rate of geosmin.

### Hardware and software used for model construction and analysis

Detailed information is listed in Supplementary Table S1.

## Results

### Model construction and characteristics analysis

**FIGURE 1:**
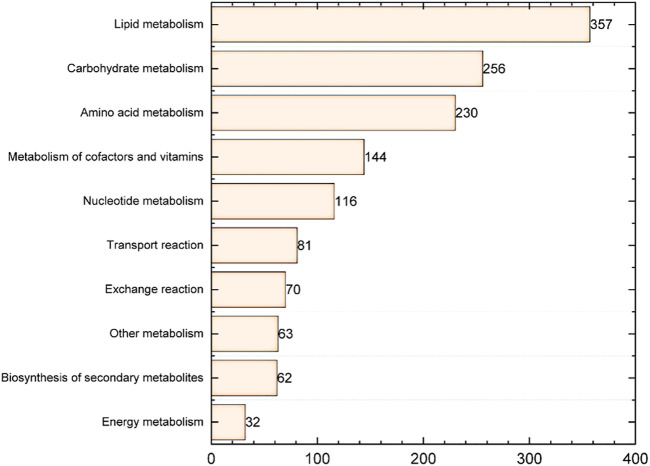
The response distribution of the metabolic subsystem in model *i*ZDZ767.

To construct the genome-scale metabolic model of *S. radiopugnans*, several steps were carried out. First, ModelSEED (Henry et al., 2010) and CarveME (Machado et al., 2018) were used to construct the draft model of *S. radiopugnans*. Then, based on the KAAS annotation (Moriya et al., 2007) results, gaps were fixed by referring to KEGG pathway maps, manually. In addition, the biomass composition of *S. radiopugnans* was identified through literature mining, which includes proteins, RNA, DNA, lipids, cell wall, and small molecules (Supplementary Material S1). Finally, the defined model was mathematized with COBRA toolbox 3.0 (Heirendt et al., 2019). The model of *S. radiopugnans* consisted of 1,411 reactions, 1,399 metabolites, and 767 genes, and was named *i*ZDZ767 (Table 1; Supplementary Material S1). Of these reactions, 88.0% were gene associated. According to the KEGG pathway maps, these reactions can be classified into 10 subsystems. Lipid metabolism, carbohydrate metabolism, and amino acid metabolism were the most common, accounting for 25.3%, 18.1%, and 16.3%, respectively (Figure 1). The gene coverage of model *i*ZDZ767 was 14.1%, which was close to the newest model *i*AA1259 of *Streptomyces coelicolor* (15.1%) (Amara et al., 2018). Cytoscape software was used to analyze the network characteristics of model *i*ZDZ767. There were 3,577 nodes and 8,706 edges in model *i*ZDZ767.

**TABLE 1:** The characteristic of model *i*ZDZ767.

**Features**	***i*ZDZ767**	***i*AA1259**
**Genome features**		
Genome size	6.1 Mb	8.59 Mb
Open reading frames	5,426	8,126
***In silico* metabolic model**		
Reactions included in the model	1,411	1914
Biochemical reactions	1,268	1,510
Transport reactions	73	195
Exchange reactions	70	209
Metabolites	1,399	1,471
Genes	767	1,259
ORF coverage (%)	14.1	15.1
**Network characteristics**		
Number of nodes	3,577	4,645
Number of edges	8,706	11427
Avg. number of neighbors	4.872	4.920
Network diameter	13	12
Network radius	7	6
Characteristic path length	4.204	4.232
Network density	0.001	0.001
Network heterogeneity	3.415	3.789
Network centralization	0.204	0.213
Connected components	2	1

### Model verification

Based on the minimal culture medium, model *i*ZDZ767 was used to simulate whether 28 carbon sources and 6 nitrogen sources could be utilized. For carbon sources, model *i*ZDZ767 achieved 82.1% (23/28) correction. For the nitrogen sources, there was 83.3% (5/6) agreement with the experimental results, which could not grow with L-Cysteine as the sole nitrogen source (Table 2). In addition, the simulated maximum growth rate (μ_max_) was 0.131 h^−1^, which was only 4.4% lower than the measured growth rate (0.137 h^−1^, Supplementary Figure S2). The essentialities of individual genes of *S. radiopugnans* were analyzed under minimal glucose medium conditions using *i*ZDZ767 by deleting each gene in turn. The genes were categorized into three classes: essential genes, partially essential genes, and non-essential genes. There were 84 genes identified as essential genes. These genes were further compared with the DEG database (Luo et al., 2021), and 97.6% of the predicted essential genes could be matched by sequence blast (identity ≥30%, e-value ≤ 1e-6, Supplementary Material S2). These results proved that model *i*ZDZ767 could predict the phenotype of *S. radiopugnans* well*.*

**TABLE 2:** The utilization of different carbon and nitrogen sources.

**Characteristics**	***In vivo***	***In silico***	**References**
**Growth on sole carbon sources**			
D-glucose	**+**	**+**	(Zhu et al., 2011)
D-Fructose	**+**	**+**	(Zhu et al., 2011)
Raffinose	**+**	**+**	(Zhu et al., 2011)
L-Arabinose	**+**	**+**	(Zhu et al., 2011)
Mannose	**+**	**+**	(Dhamodharan et al., 2019)
Lactose	**+**	**+**	(Dhamodharan et al., 2019)
Trehalose	**+**	**+**	(Dhamodharan et al., 2019)
Melibiose	**−**	**+**	(Dhamodharan et al., 2019)
Acetate	**+**	−	(Dhamodharan et al., 2019)
Inositol	**−**	**−**	(Mao et al., 2007)
Mannitol	**+**	**+**	(Mao et al., 2007)
L-Rhamnose	**+**	**+**	(Mao et al., 2007)
Sucrose	**+**	**+**	(Mao et al., 2007)
Xylitol	**+**	**+**	(Mao et al., 2007)
D-xylose	**+**	**+**	(Mao et al., 2007)
Citrate	**+**	**+**	(Mao et al., 2007)
L-Arginine	**+**	**+**	(Dhamodharan et al., 2019)
L-Alanine	**+**	**+**	(Dhamodharan et al., 2019)
L-Aspartate	**+**	**+**	(Dhamodharan et al., 2019)
Glycine	**+**	**+**	(Dhamodharan et al., 2019)
L-Histidine	**−**	**+**	(Dhamodharan et al., 2019)
L-Lysine	**+**	**+**	(Dhamodharan et al., 2019)
L-phenylalanine	**+**	**+**	(Dhamodharan et al., 2019)
L-Tryptophan	**+**	−	(Dhamodharan et al., 2019)
L-Methionine	**+**	**−**	(Dhamodharan et al., 2019)
L-Isoleucine	**−**	**−**	(Dhamodharan et al., 2019)
L-Valine	**−**	**−**	(Dhamodharan et al., 2019)
L-Asparagine	**−**	**−**	(Dhamodharan et al., 2019)
**Growth on sole nitrogen sources**			
NH_4_^+^	**+**	**+**	This study
KNO_3_	**+**	**+**	This study
Urea	**+**	**+**	This study
L-Cysteine	**+**	**−**	(Mao et al., 2007)
L-phenylalanine	**+**	**+**	(Santhanam et al., 2012)
L-Threonine	**+**	**+**	(Santhanam et al., 2012)

### The optimization of culture condition with *i*ZDZ767

**FIGURE 2:**
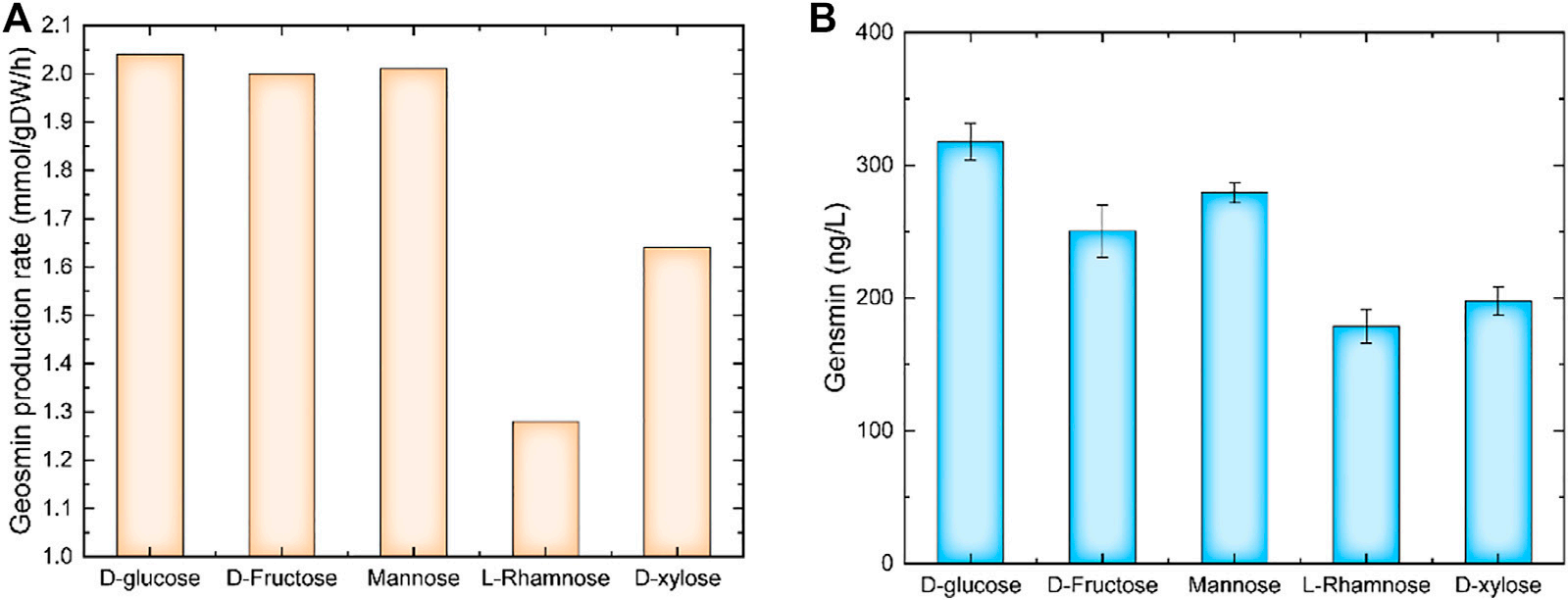
The effect of carbon sources on geosmin production. **(A)** Simulation results of different carbon sources. **(B)** Experimental results of different carbon sources.

**FIGURE 3:**
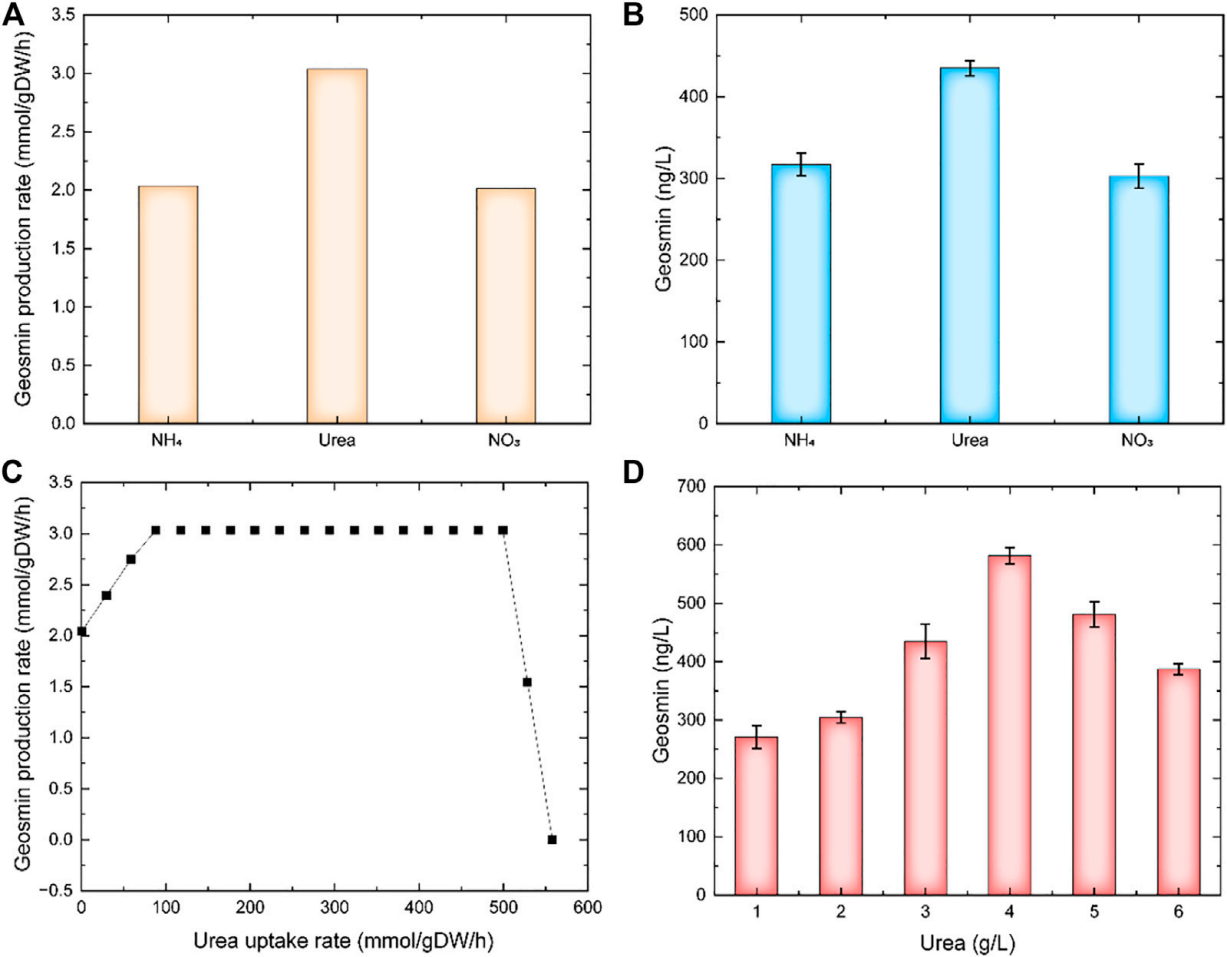
The effect of nitrogen sources on geosmin production. **(A)** simulation results of different nitrogen sources. **(B)** Experimental results of different nitrogen sources. **(C)** Robustness analysis results of urea uptake rate. **(D)** Effect of urea concentration on geosmin production.

The carbon source was a key factor for cell growth and product synthesis. Using model *i*ZDZ767, the effect of different carbon sources, such as D-glucose, D-Fructose, Mannose, L-Rhamnose, and D-xylose was predicted. Of these selected carbon sources, D-glucose was the most suitable, the GPR was 2.04 mmol/gDW/h (Figure 2A). The experimental results show that when D-glucose was used as a carbon source, the yield of geosmin was 317.5 ng/L, which was higher than others (Figure 2B). Similarly, three types of nitrogen sources were used for simulation. The model predicted that when urea was used as a nitrogen source, the GPR was 3.03 mmol/gDW/h, which was 48.9% and 50.7% higher than NH_4_^+^ and NO_3_^−^ (Figure 3A). Compared with the experiments, the geosmin yield was 435.1 ng/L, which agreed with the simulation (Figure 3B). In addition, a robustness analysis algorithm was used to analyze the effect of the urea uptake rate on the geosmin production rate. The simulated results showed that with the increase in the urea uptake rate, the GPR would first increase to the maximum value, then remain stable until the urea uptake rate was over 500 mmol/gDW/h. Finally, the GPR would decrease to 0, which means that the suitable urea uptake rate should be 88.38 mmol/gDW/h (Figure 3C). When we controlled the addition of urea at different levels, the experiment results proved that 4 g/L urea could achieve a maximum geosmin production of 581.6 ng/L (Figure 3D).

### Identification of the potential geosmin overproduction targets with *i*ZDZ767

**FIGURE 4:**
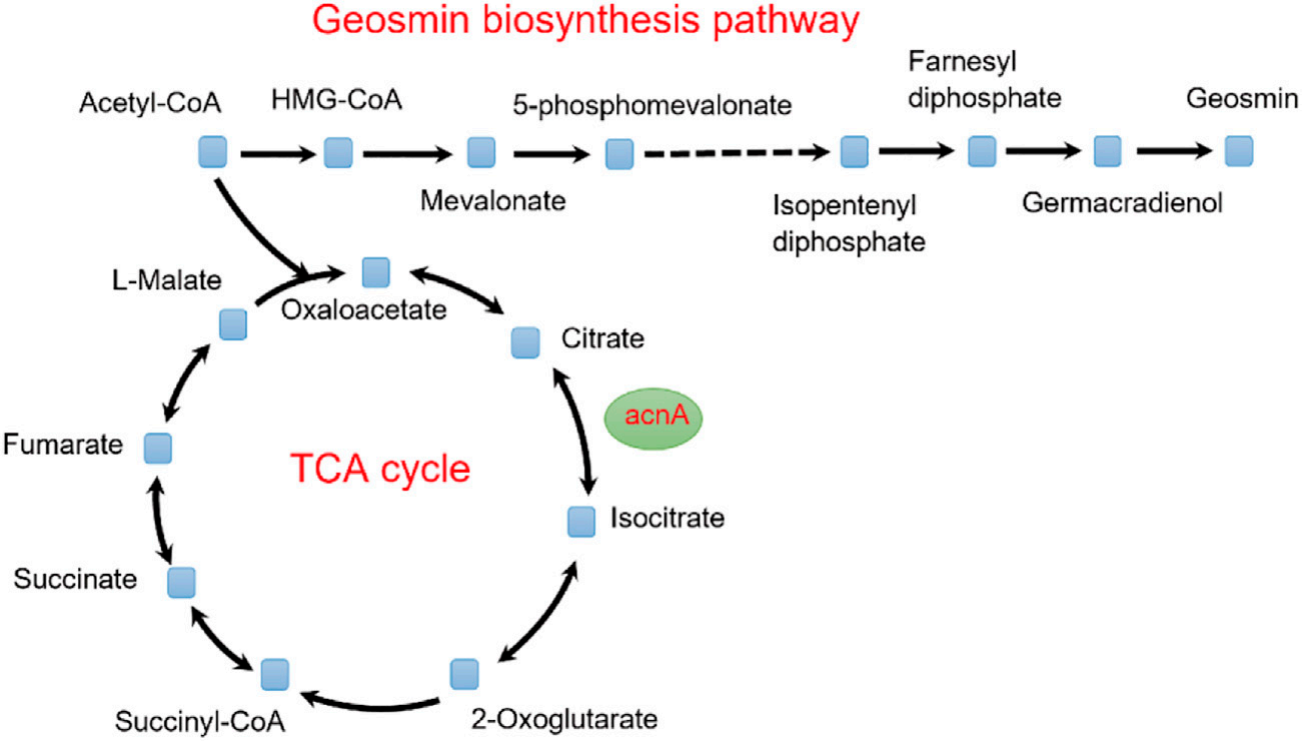
Effect of the TCA cycle on the synthesis of geosmin.

To identify potential targets for the improvement of geosmin production, the OptForce algorithm was used (Ranganathan et al., 2010). According to the predicted results, a total of 29 genes were identified as the targets, including seven upregulation, six downregulation, and 16 knockout targets (Supplementary Material S3). According to the function of each gene, these targets can be classified into four types: precursor accumulation, geosmin biosynthesis, by-product elimination, and energy supplement. For the geosmin synthesis pathway, the *geoA* gene, which encodes geosmin synthase, catalyzing the synthesis of geosmin from farnesyl diphosphate, should be upregulated (Shen et al., 2021). For by-product elimination, to accumulate more geosmin, the *acnA* gene (Aconitate hydratase A) should be downregulated to decrease the carbon flux of the TCA cycle (Figure 4). Similarly, the *fabD* gene [(acyl-carrier-protein) S-malonyltransferase] should also be downregulated to limit the flux of fatty acids synthesis. For energy supplements, the *nuo* gene (NADH-quinone oxidoreductase) was predicted to be knocked out so that more NADH could be supplied for geosmin synthesis.

## Discussion

Geosmin is a common pollutant and is widely recognized by the public, but this is not the case when studying some biological systems and organisms. Toxicological studies have shown that a certain concentration of geosmin could inhibit the growth of *Salmonella typhimurium* and sea urchin embryos (Dionigi et al., 1993; Nakajima et al., 1996). This provides a new direction for the study of how to inhibit pathogens. At the same time, researchers have also found that geosmin has a potential effect on genotoxicity. Geosmin is only mildly toxic at extremely high concentrations, far exceeding the actual level in the environment (Silva et al., 2015). Some researchers have found that geosmin, at a concentration of 50–5000 ng/L, can increase the body length and change the growth-related genes of zebrafish (Zhou et al., 2020).

*S. radiopugnans* can be screened from radiation-contaminated soil and, although not widely studied, are capable of producing geosmin in large quantities. However, phenotypes of *S. radiopugnans* are difficult to study due to their complex cellular metabolism and regulatory mechanisms. Therefore, by manually refining the first genome-scale metabolic network model (*i*ZDZ767) of *S. radiopugnans*, we analyzed the synthesis mechanism of geosmin and identified the key targets of geosmin synthesis based on the model, which provided a basis for further study of the synthesis of geosmin and the internal mechanism of *S. radiopugnans*.

Traditional model construction methods are mainly divided into automatic construction and manual construction. Among them, automatic construction automatically obtains the GSMM of the target strain or plant by uploading the genomic data to the existing tools. To date, researchers have developed many tools, such as ModelSEED (Flowers et al., 2018; Seaver et al., 2021), COBRA (Dal’molin et al., 2014; King et al., 2015), and RAVEN (Wang et al., 2018), for the automatic construction of models. The advantage of this construction method is that the model can be built in a short time, but the accuracy of the model is low and its applicability is not strong. The manual construction of this method mainly depends on the results of genome annotation, combined with the metabolic pathway of KEGG, it collates information, such as the genes and metabolites of each reaction, and manually adds it to the model. Considering the problems of traditional modeling methods, we used a semi-automatic modeling method. This method integrates the first two methods, first obtaining a coarse model through the automated construction tool, then manually refining the model so that a more accurate model can be obtained. Based on the automatic construction of the ModelSEED database and CarveME (Machado et al., 2018), combined with the complete metabolic pathway in KEGG, we added the missing reaction to the model and added the known metabolic pathway of geosmin to the model and, thus, manually refined a genome-scale metabolic network model of *S. radiopugnans*. Based on model *i*ZDZ767, a series of strategies for improving geosmin were proposed. Although *S. radiopugnans* can make good use of microbial fermentation to produce geosmin, its metabolic network is complex, and the fermentation experiment period is long. It depends on repeated experiments to increase the production of geosmin, and the economic cost is high. Model *i*ZDZ767 can predict the effects of carbon and nitrogen sources on the synthesis rate of geosmin well and provide directions for optimizing the culture conditions of *S. radiopugnans*. At the same time, the model is used to analyze the algorithm to predict the key targets for improving the geosmin synthesis rate. Through the analysis of these key targets, we found that the key reactions affecting the synthesis of geosmin are mainly divided into two types. One type of reaction is related to the synthesis of geosmin itself, while the other is related to the growth and reproduction of *S. radiopugnans*. No matter which type of reaction is upregulated, downregulated, or knocked out, the synthesis of geosmin can be effectively improved. Although the effectiveness of these regulation methods has not been proven, they provide the opportunity to study geosmin production by fermentation. In summary, model *i*ZDZ767 is a powerful tool for analyzing and predicting the metabolic pathways and yields of various products in *S. radiopugnans*, which provides convenient conditions for us to study *S. radiopugnans* in the field of systems biology.

## Data Availability

Publicly available datasets were analyzed in this study. This data can be found here: https://www.uniprot.org/taxonomy/403935.
