# Supplementary material for: Construction and application of the genome-scale metabolic model of Streptomyces radiopugnans
Source: Front Bioeng Biotechnol. 2023 Feb 17;11:1108412. doi: 10.3389/fbioe.2023.1108412 (PMC9982006; doi:10.3389/fbioe.2023.1108412)
Supplement: Supplementary file 1 [file Table1.DOCX]

Table S1 Detailed information of hardware and software used in this study

| **Hardware** |  |
| --- | --- |
| High performance server | Dell ThinkSystem SR650 |
| CPU | 2 * Intel Xeon Gold 5220R |
| GPU | NVIDIA Quadro RTX 5000 16GB |
| Memory | 8 * 32GB TruDDR4 2933MHz |
| **Software** |  |
| Operating system | Centos 7.6 |
| MATLAB | 2017b |
| COBRA toolbox | 3.0 |
| Gurobi Optimizer | 9.5.1 |
| libSBML | 5.16.0 |
| SBMLToolbox | 4.1.0 |
| Cytoscape | 3.9.1 |
| Origin | 2023 |
