# Supplementary figures and images for: Construction and application of the genome-scale metabolic model of Streptomyces radiopugnans
Source: Front Bioeng Biotechnol. 2023 Feb 17;11:1108412. doi: 10.3389/fbioe.2023.1108412 (PMC9982006; doi:10.3389/fbioe.2023.1108412)

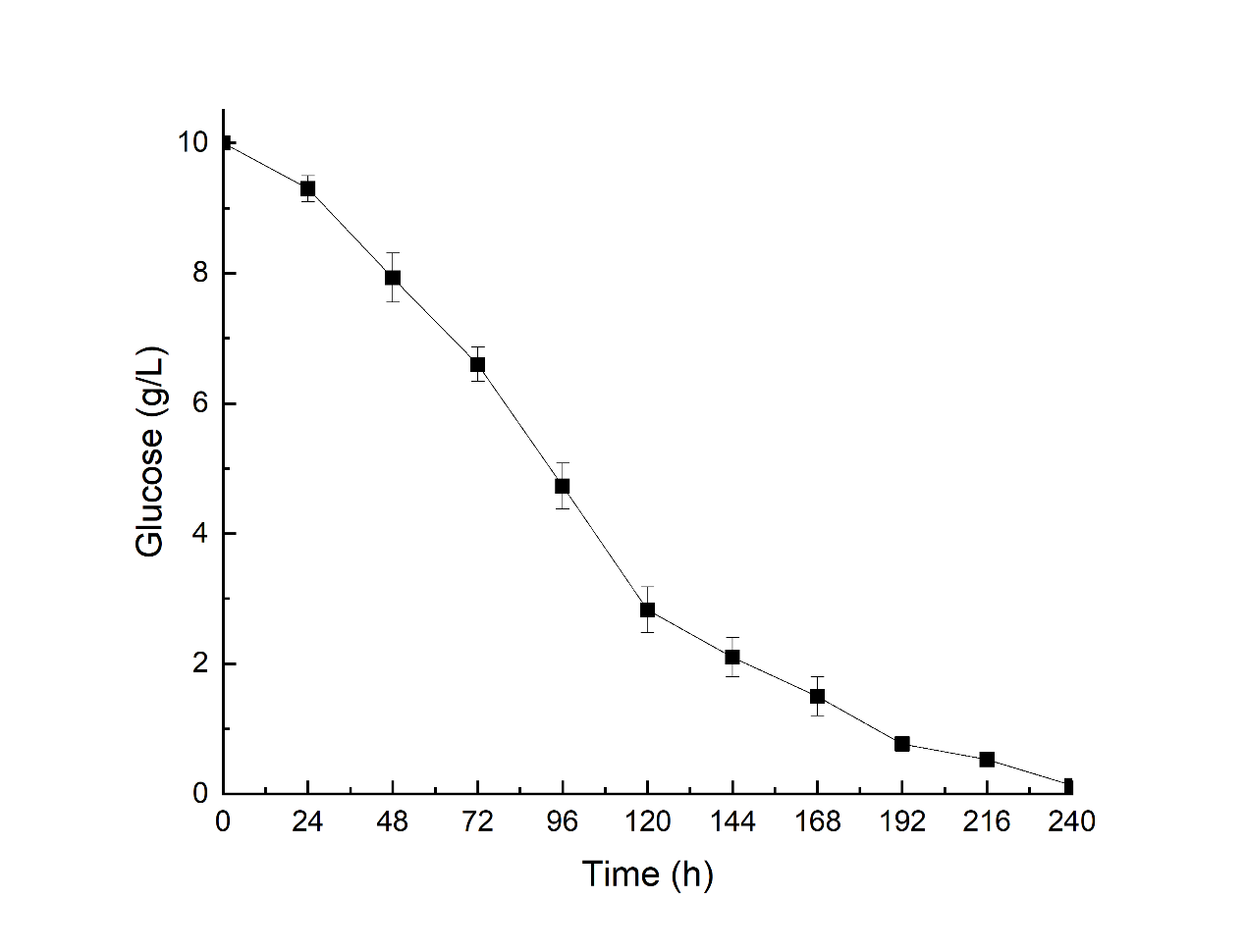


Fig. S3 The glucose consumption rate of *S. radiopugnans.*

Supplement: Supplementary file 4 [file DataSheet3.docx]

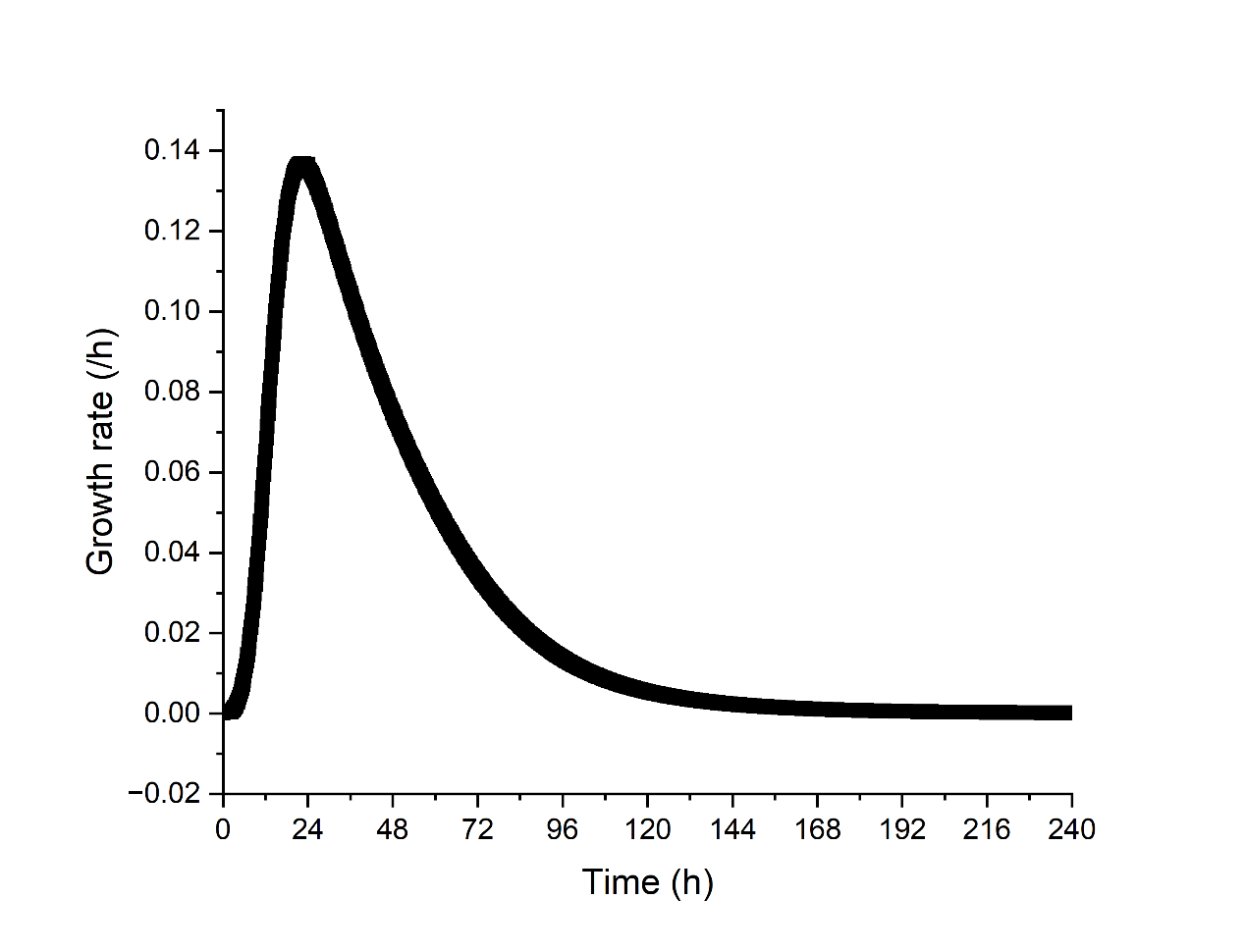


Fig. S2 The calculated specific growth rate of *S. radiopugnans.*

Supplement: Supplementary file 5 [file DataSheet2.docx]

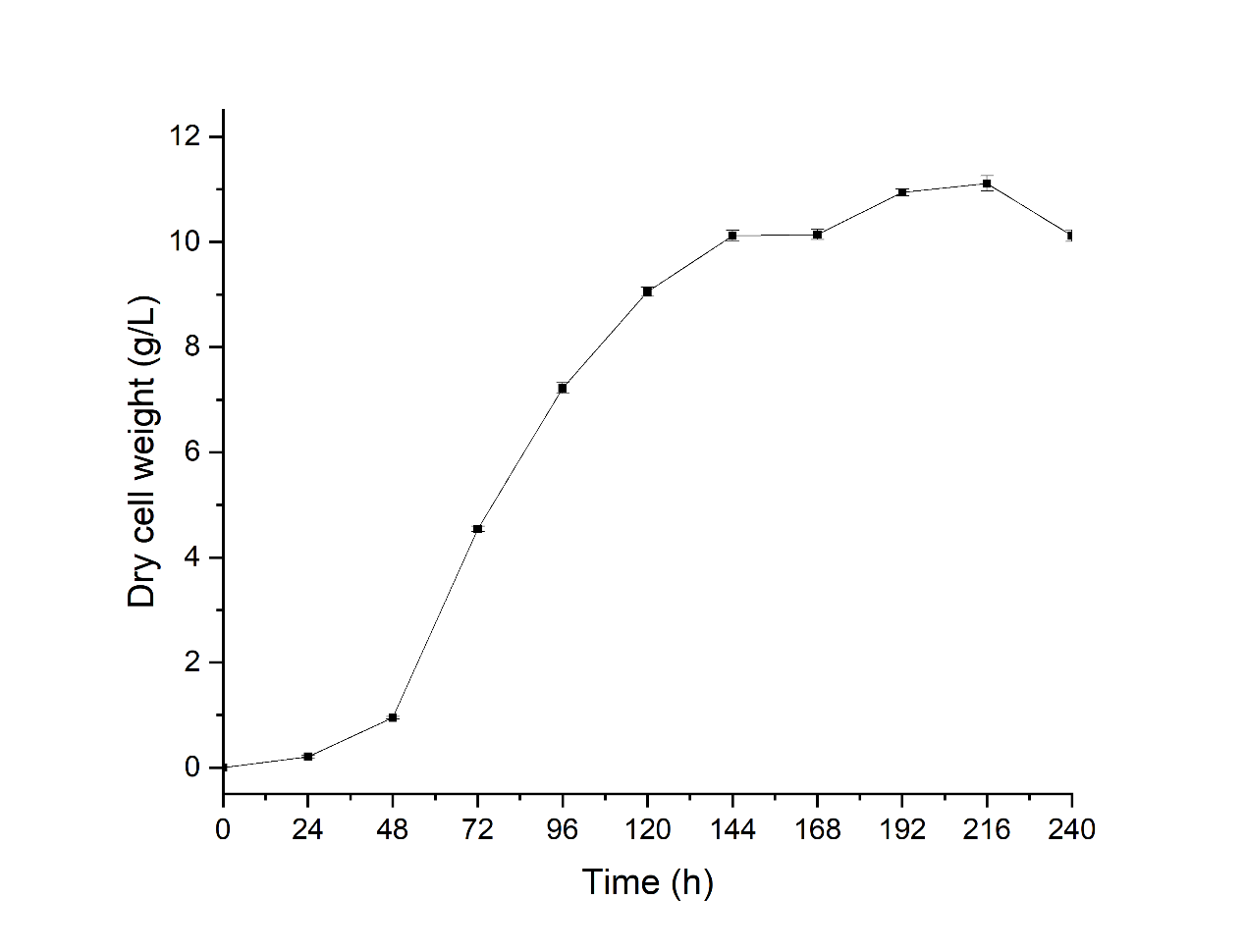


Fig. S1 The growth curve of *S. radiopugnans.*

Supplement: Supplementary file 7 [file DataSheet1.docx]
